# Preoperative Enterosignatures Predict Surgical Site Infections After Abdominal Surgery

**DOI:** 10.1093/ofid/ofaf549

**Published:** 2025-09-03

**Authors:** Simone N Zwicky, Daniel Spari, Daniel Rodjakovic, Hugo Guillen-Ramirez, Bahtiyar Yilmaz, Guido Beldi

**Affiliations:** Department of Visceral Surgery and Medicine, Inselspital, Bern University Hospital, University of Bern, Bern, Switzerland; Department of Visceral Surgery and Medicine, Inselspital, Bern University Hospital, University of Bern, Bern, Switzerland; Department of Visceral Surgery and Medicine, Inselspital, Bern University Hospital, University of Bern, Bern, Switzerland; Department of Visceral Surgery and Medicine, Inselspital, Bern University Hospital, University of Bern, Bern, Switzerland; Department of Visceral Surgery and Medicine, Inselspital, Bern University Hospital, University of Bern, Bern, Switzerland; Department of Visceral Surgery and Medicine, Inselspital, Bern University Hospital, University of Bern, Bern, Switzerland

**Keywords:** enterosignatures, microbiota profiling, surgical site infection

## Abstract

**Background:**

The relationship between preoperative intestinal microbiota composition and the development of surgical site infections (SSIs) following abdominal surgery is not well understood. The aim of this study was to characterize the preoperative rectal microbiota using the novel concept of enterosignatures (ESs) in patients undergoing abdominal surgery and assess their association with SSIs.

**Methods:**

In this prospective cohort study, preoperative rectal microbiota from 133 patients undergoing elective abdominal surgery was profiled using 16S rRNA sequencing. ESs were calculated using high-quality genus-level taxonomy, simplifying complex microbial compositions into 5 generalizable patterns: *Bacteroides-, Firmicutes-, Prevotella-, Bifidobacterium-,* or *Escherichia*-dominated profiles.

**Results:**

A total of 519 bacterial species were identified within the 133 patients. The *Firmicutes* ES was found to be a significant risk factor for SSIs, while the *Prevotella* ES was associated with a reduced risk of SSIs. Combining these into the *Firmicutes*-to-*Prevotella* ES ratio (ES-Firm-Prev ratio) yielded a stronger association with SSIs (noSSI: median [interquartile range {IQR}] log ES-Firm-Prev ratio, 0.21 [−0.43 to 1.33]; vs SSI: median [IQR] log ES-Firm-Prev ratio, 8.24 [2.17 to 8.5]; *P* = .001). Machine learning and logistic regression models confirmed the ES-Firm-Prev ratio to be a significant, independant predictor of SSIs (odds ratio, 1.35; 95% CI, 1.09–1.66; *P* = .005).

**Conclusions:**

The ES-Firm-Prev ratio is a robust, independent predictor of SSIs in patients undergoing abdominal surgery and may serve as a novel biomarker to identify high-risk patients preoperatively.

## Graphical Abstract

**Graphical Abstract:**
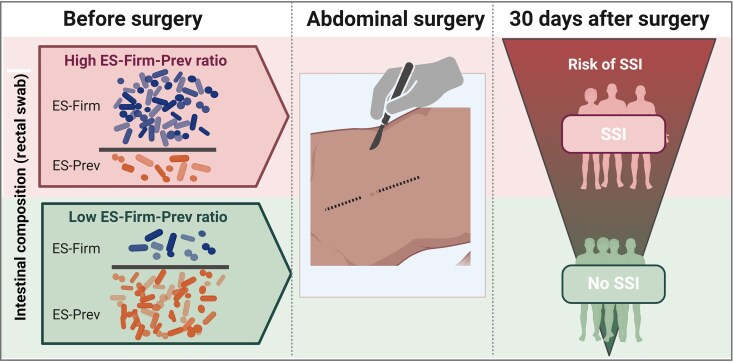

Each year, >300 million surgeries are performed worldwide [1], with surgical site infections (SSIs) ranking among the most common hospital-acquired infections [2, 3]. The incidence of SSIs varies considerably by country income level [4] and surgery type, with abdominal procedures exhibiting particularly high rates [4, 5]. SSIs increase the duration of hospital stay, the rate of reoperations, and the risk for intensive care unit treatments, imposing substantial burdens on patients and health care systems [6, 7]. Multiple patient- and procedure-related risk factors for SSIs have been identified [8, 9]. Despite addressing them, as well as applying established antiseptic measures in industrialized countries, the rate of SSIs after abdominal surgery remains about 10% [4, 5, 10].

The intestinal microbiota composition is essential for maintaining the intestinal barrier and providing resistance to infection [11–15]. Disruptions in intestinal microbiota composition are associated with increased rates of bacterial translocation and infections [11, 14–16]. In critically ill patients, rectal enrichment of *Enterobacteriaceae* is associated with nosocomial infections [15], and in patients undergoing allogeneic hematopoietic stem cell transplantation, intestinal dominance of *Enterococcus* has been linked with risk of bacteremia [14].

Animal models suggest that microbiota disruptions lead to subsequent SSIs [17] and impair postsurgical survival [18]. Yet, human studies examining associations between preoperative intestinal microbiota composition and surgical outcomes remain limited. Some studies suggest that *Acinetobacter* or *Hafnia* contribute to anastomotic leakage [19], while increased levels of individual species such as *Akkermansia muciniphila, Butyricimonas* sp. [20], or specific community structures [21] are reported to correlate with postoperative complication risk. Furthermore, ileal abundance of *Fusobacterium* and certain *Firmicutes* have been associated with SSIs [22]. However, these studies struggle to generalize their findings due to the extensive individual variation in intestinal microbiota composition [19–22]. Additionally, there is a lack of focused research on preoperative microbiota composition and SSI risk.

The human intestinal microbiota is a complex community of >1000 bacterial species and is unique in each patient [23]. Breaking it down to universally applicable patterns might therefore be beneficial for clinical application. Recently, Frioux et al. identified 5 generalizable enterosignatures (ESs), dominated by either *Bacteroides* (ES-Bact), *Bifidobacterium* (ES-Bifi), *Escherichia* (ES-Esch), *Prevotella* (ES-Prev), or *Firmicutes* (ES-Firm) [24]. Typically, 2 or more ESs are required to adequately capture the variation within the microbial community in an intestinal sample [24]. The novel concept of ESs characterizes coexisting bacterial communities and allows generalization to a variety of populations and nations. The ES model [24] has shown stronger associations with clinical variables compared with the older enterotype model [25].

ESs thereby have the potential to reveal important relationships between intestinal microbiota and clinical outcomes. However, it is unknown if there is an association between preoperative ESs and postoperative complications such as SSIs. In this prospective study, we aim to investigate the association between preoperative ESs and occurrence of SSIs after elective abdominal surgery.

## METHODS

### Study Population and Design

We conducted a prospective cohort study, including 138 patients undergoing abdominal surgery from December 1, 2020, to December 31, 2021. Inclusion criteria were (1) age ≥18 years, (2) general consent present, and (3) elective abdominal surgery, (4) including colorectal resection, pancreatic resection, liver resection, and gastric bypass. There were no exclusion criteria. All patients were hospitalized and were followed up for 30 days after surgery for occurrence of SSIs (100% of participants). Data were analyzed from June 2022 to May 2024. Five patients without preoperative rectal swabs were excluded, and 16S rRNA sequencing was performed on swabs from 133 patients. High-quality genus-level taxonomy from 124 patients was used to calculate ESs. Patients with good model fit (>0.4) were included for further analysis. The study was approved by the Kantonale Ethikkomission Bern, Switzerland (ethical approval 2019-00576, NCT04096885). All patients signed written informed consent. The study follows the STROBE reporting guidelines.

### Outcomes and Variables

The primary outcomes were SSIs within 30 days after surgery, according to the definition of the Centers for Disease Control and Prevention criteria [26]. These criteria classify SSIs as infections of the skin and subcutaneous tissue (superficial incisional), deep soft tissues (deep incisional), or organ/space tissues, for example, intra-abdominal infection (organ/space) [26]. Diagnosis requires, in addition to the infected site, meeting a combination of specific criteria for each infection type, such as purulent drainage, organism identification, opening of the incision with signs like pain, tenderness, or fever, or a physician diagnosis, with some types requiring confirmation through anatomical exam, histopathologic exam, or imaging tests [26]. SSIs were evaluated by trained study nurses following the Swissnoso SSI surveillance guide, which aligns with internationally recognized standards used by the US National Healthcare Safety Network (NHSN) [27, 28]. Baseline covariates and therapeutic and surgical features were extracted from medical records. Antibiotic therapy was defined as use of antibiotics for >48 hours within 12 weeks before surgery, 1-time surgical antibiotic prophylaxis before surgery was therefore not included. Bowel preparation was administered to a subset of patients in the form of 2 doses of a laxative (1 in the morning and 1 in the evening the day before surgery).

### Sample Collection and 16S rRNA Sequencing

Standardized rectal mucosa swabs were collected directly before surgery (after intubation, before antibiotic prophylaxis) and streaked on filter paper (Whatman FTA cards). Samples were stored at room temperature for subsequent use. Six 1-mm pouches were taken from 1 filter paper and put into polymerase chain reaction tubes. Pouches were washed using QIAcard FTA Wash Buffer according to the manufacturer's protocol, and sequencing of V5/V6 of the 16S rRNA gene was performed as described elsewhere, starting with the pouches as input DNA material [18].

Raw reads were processed on the UBELIX cluster at the University of Bern using the QIIME2 pipeline as described elsewhere [18, 29, 30].

### Enterosignature Calculation

The relative abundance genus-level taxonomy table was reclassified using GTDB, which is required as input for calculating the ESs [24, 31]. Briefly, the non-negative matrix factorization (NMF) algorithm is applied to calculate the matrices W and H. The matrices are normalized to get the weight of genera in ESs (W matrix) and the relative abundance of ESs in samples (H matrix) [24].

### Machine Learning Model Implementation, Evaluation, and Visualization

We trained several classifiers using leave-1-out cross-validation: Ridge, ExtraTrees, XGBoost, Random Forest, K-Nearest Neighbors (KNN), AdaBoost, Decision Tree, and Naive Bayes. This approach involved training each model on the data set minus 1 sample and then testing it on the excluded sample, iterated such that each sample served as a test sample exactly once. Bacterial signatures were normalized at each iteration to avoid data leakage [32]. An F1-score was calculated for each model.

To elucidate the contribution of each feature to the prediction of SSIs, we employed SHapley Additive exPlanations (SHAP) [33] values using XGBoost trained on the whole data set. We plotted the features with the highest average absolute SHAP values, displaying each feature's SHAP value in a scatter plot where the color intensity indicates the normalized feature values. A higher SHAP value indicates higher probability of an SSI outcome.

### Statistical Analysis

All statistical analyses were performed in R (version 4.3.3), except for machine learning, which was conducted in Python (version 3.9.0). Only 2-sided tests were used. A Wilcoxon rank-sum test compared non–normally distributed continuous data, while Fisher's exact test was used for categorical data. A logistic regression model was calculated using the *glmer()* function from the “lme4” R package using type of surgery as a random variable. To compare >2 groups, the Kruskal-Wallis test followed by a pairwise Wilcoxon rank-sum test with Benjamini-Hochberg (BH) correction was used. Pairwise correlations were determined using Spearman's rank correlation coefficient and visualized with a hierarchical clustering heatmap, where row and column dendrograms depict hierarchical relationships based on Euclidean distance and average linkage method. Calculation of beta diversity and principal component analysis, Procrustes, enrichment, and PICRUSt2 analysis, was performed as described elsewhere [18].

## RESULTS

### ESs are Calculated From Bacterial Genera Identified by 16S rRNA Sequencing

**Figure 1.**
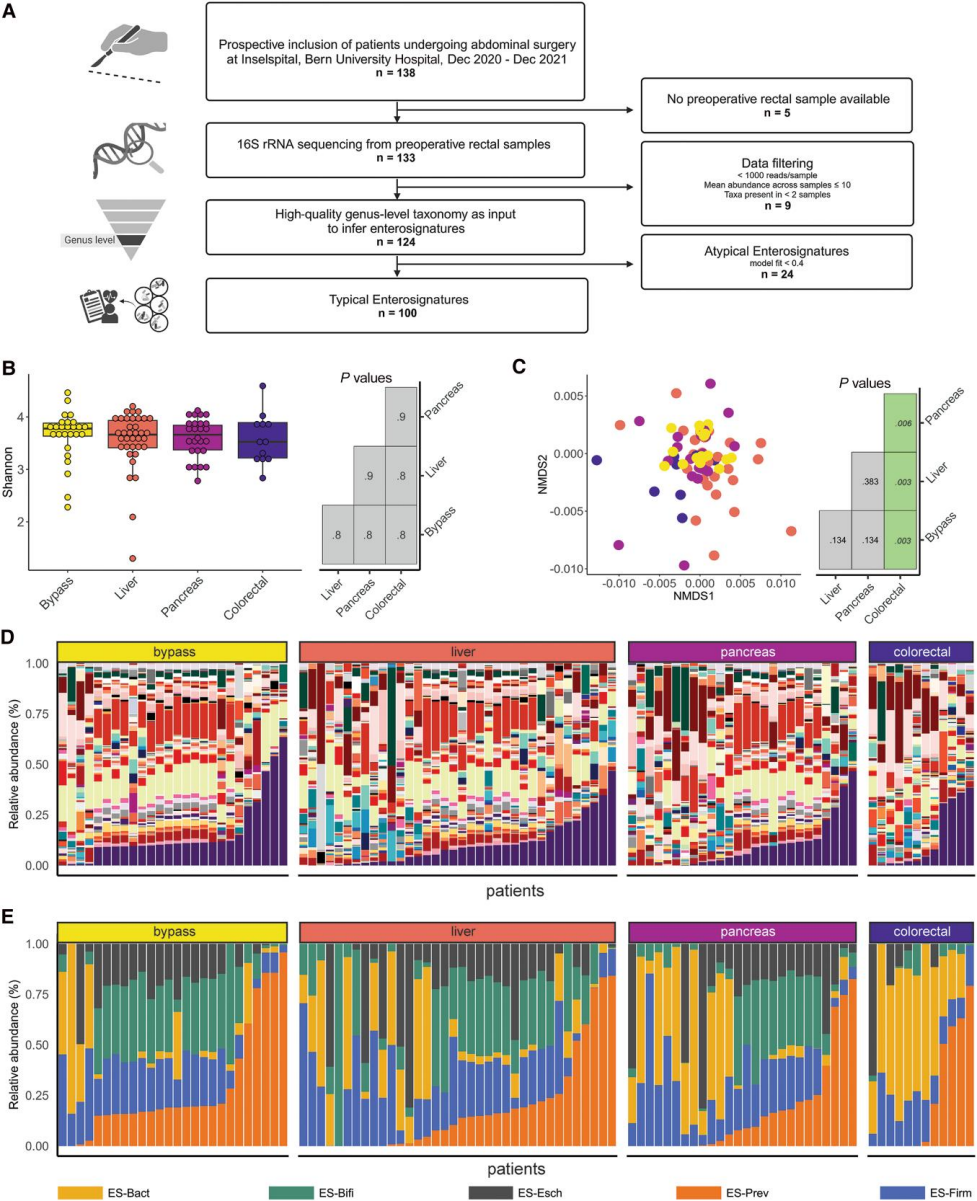
*A*, Flowchart depicting the study cohort. *B*, Sample diversity (alpha diversity, Shannon). *C*, Community diversity (beta diversity, Bray-Curtis). *D*, Taxonomic composition on the genus level. E, Composition of ESs for each patient separated according to type of surgery. Abbreviations: Bact, *Bacteroides*; Bifi, *Bifidobacterium*; Dec, December; ES, enterosignature; Esch, *Escherichia*; Firm, *Firmicutes*; NMDS, nonmetric multidimensional scaling; Prev, *Prevotella*.

The study flowchart is shown in Figure 1*A*. To establish a clinically practical sampling method, we first compared rectal swabs streaked on filter paper and stored at room temperature with directly frozen swabs (Supplementary Figure 1A–G). No differences in diversities or microbiota composition were observed, confirming the reliability of this easy-to-use method. We obtained standardized rectal swabs directly before surgery (after intubation for the procedure). We then performed 16S rRNA sequencing on rectal swabs obtained from 133 patients, identifying 519 bacterial species. Quality control (criteria in Figure 1*A*) resulted in 50 high-quality genus-level taxa from 124 patients, which were used to calculate ESs (enterosignatures.quadram.ac.uk). In accordance with the original publication, only patients with good quality of ES calculation (model fit >0.4) were included for further analysis (n = 100) [24].

Sample diversity (alpha diversity; Shannon Index) was not significantly different across types of surgery (Figure 1*B*). Community diversity (beta diversity; Wunifrac-based nonmetric multidimensional scaling [NMDS]) was significantly different in patients with colorectal surgery when compared with other surgery types (Figure 1*C*). No significant differences in sample and community diversity were observed between the noSSI and SSI groups (Supplementary Figure 1*H* and *I*). Furthermore, no significant associations between SSIs and the relative abundance of individual genus-level taxa were identified using MaAsLin2 (Multivariate Association with Linear Models 2) [34]. Comparison of the genus-level bacterial relative abundances (Figure 1D;  Supplementary Figure 2) with the relative abundances of ESs (Figure 1*E*) highlights a clear reduction of complexity using ESs. Figure 1A was created with BioRender.com.

### The ES-Firm-Prev Ratio Serves as an Independent Preoperative Risk Factor for the Development of SSIs

**Figure 2.**
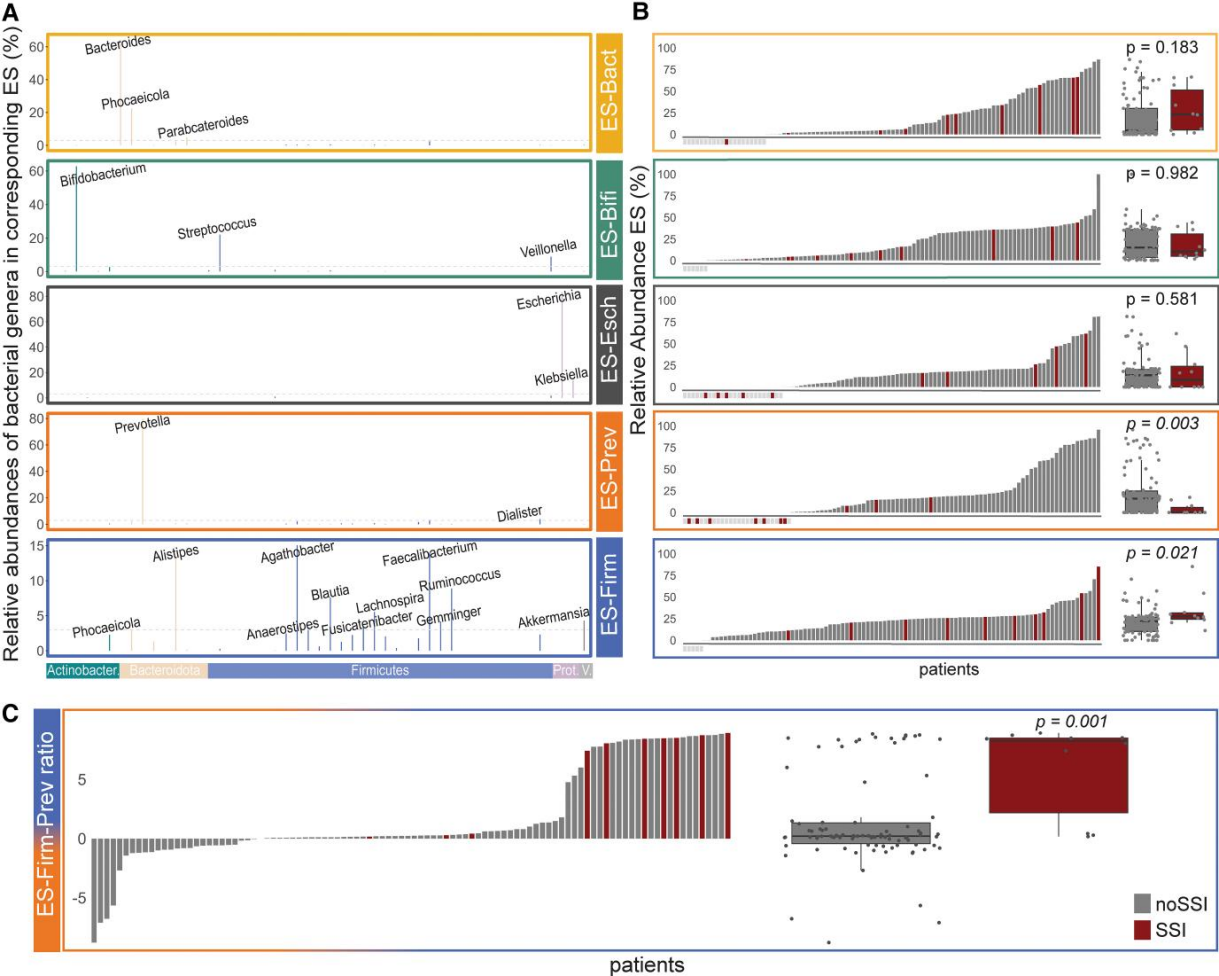
*A*, Relative abundance of dominant genera contributing to each ES. *B*, Relative abundance of each ES for patients without and with SSI. *C*, ES-Firm-Prev ratio for patients without and with SSI. Abbreviations: Actinobacter., Actinobacteria; Bact, *Bacteroides*; Bifi, *Bifidobacterium*; ES, enterosignature; Esch, *Escherichia*; Firm, *Firmicutes*; Prot., Proteobacteria; SSI, surgical site infection; V., Verrucomicrobiota.

Each of the 5 ESs represents a weighted combination of genera (Figure 2*A*). The ESs are mostly composed of the following genera: ES-Bact (*Bacteroides* and *Phocaeicola*), ES-Bifi (*Bifidobacterium* and *Streptococcus*), ES-Esch (*Escherichia* and *Klebsiella*), ES-Prev (*Prevotella*), and ES-Firm (*Firmicutes* genera and *Alistipes*). Detailed insights on ES compositions and functions have been provided by Frioux et al. [24].

Next, we assessed the importance of each ES on the occurrence of SSIs (Figure 2*B*, Table 1). The relative abundance of ES-Firm was significantly higher in patients with SSI compared with patients without SSI (noSSI: median [interquartile range {IQR}], 21.9 [10.3 to 26.7]; vs SSI: median [IQR], 27.7 [24.3 to 31.7]; *P* = .021). Conversely, the relative abundance of ES-Prev was significantly lower in patients with SSI compared with noSSI patients (noSSI: median [IQR], 16.0 [1.0 to 24.8]; vs SSI: median [IQR], 0.0 [0.0 to 5.8]; *P* = .003). Given the strong effect, we combined the 2 ESs with significant positive (ES-Firm) and negative (ES-Prev) associations into a new parameter: the ES-Firm-Prev ratio. We found an even more significant association between the ES-Firm-Prev ratio and SSIs (noSSI: median [IQR] log ES-Firm-Prev ratio, 0.21 [−0.43 to 1.33]; vs SSI: median [IQR] log ES-Firm-Prev ratio, 8.24 [2.17 to 8.5]; *P* = .001) (Figure 2*C*). All SSI patients in our cohort (n = 10, 10%) had a positive ES-Firm-Prev-ratio. The ES-Firm-Prev ratio combined with type of surgery revealed no significant differences among the different surgery types (Supplementary Figure3). The ESs of the noSSI and SSI groups are depicted in Supplementary Figure 3*B*.

**Table 1.** Association of Baseline Characteristics and Enterosignatures With Surgical Site Infection

	Surgical Site Infection		*P* Value^b^	LR^c^	OR (95% CI)
Characteristics	No (n = 90)	Yes (n = 10)			
Age, median [IQR], y	66.5 [50.0 to 77.5]	77.5 [73.5 to 80.0]	.01*	…	…
Sex					
Male, No. (%)	41 (45.6)	6 (60.0)	.509	…	…
BMI, median [IQR], kg/m^2^	27.10 [24.2 to 34.9]	27.45 [23.3 to 29.6]	.280*	…	…
Comorbidities, No. (%)					
Diabetes	9 (16.1)	0 (0.0)	.580	…	…
Cancer	49 (54.4)	6 (60.0)	1.00	…	…
ASA score 2	25 (28.4)	3 (30.0)	1.00	…	…
ASA score 3	62 (70.5)	7 (70.0)	1.00	…	…
ASA score 4	1 (1.1)	0 (0.0)	1.00	…	…
Medication, No. (%)					
Antihypertensive drugs	29 (50.0)	4 (66.7)	.673	…	…
Lipid-lowering drugs	17 (30.9)	3 (50.0)	.384	…	…
PPI	27 (50.0)	3 (50.0)	1.00	…	…
Preoperative antibiotic therapy^a^	4 (4.4)	1 (10.0)	.416	…	…
Bowel preparation	2 (2.2)	1 (10.0)	.273	…	…
Surgery					
Duration of surgery, median [IQR], min	125.5 [72.0 to 243.8]	308.0 [286.0 to 409.0]	.001*	0.042	1.01 (1.00–1.02)
Laparotomy, No. (%)	30 (33.3)	9 (90.0)	.001	…	…
Type of surgery, No. (%)					
Gastric bypass	26 (28.9)	0 (0.0)	.060	…	…
Colorectal	11 (12.2)	1 (10.0)	1.000	…	…
Liver resection	35 (38.9)	1 (10.0)	.090	…	…
Pancreatic resection	18 (20.0)	8 (80.0)	<.001	…	…
ES relative abundance, median [IQR], %					
Bact	4.7 [0.7 to 30.1]	23.2 [5.2 to 51.3]	.183*	…	…
Bifi	14.8 [3.4 to 35.8]	10.0 [4.7 to 30.8]	.982*	…	…
Esch	13.6 [0.9 to 20.2]	8.1 [0.0 to 23.9]	.581*	…	…
Prev	16.0 [1.0 to 24.8]	0.0 [0.0 to 5.8]	.003*	…	…
Firm	21.9 [10.3 to 26.7]	27.7 [24.3 to 31.7]	.021*	…	…
Log Firm-Prev ratio	0.21 [−0.43 to 1.33]	8.24 [2.17 to 8.50]	.001*	0.005	1.35 (1.09–1.66)

Abbreviations: ASA, American Society of Anesthesiologists; Bact, *Bacteroides*; Bifi, *Bifidobacterium*; BMI, body mass index; ES, enterosignature; Esch, *Escherichia*; Firm, *Firmicutes*; IQR, interquartile range; Log, logarithm; LR, logistic regression; NAs, not applicable; OR, odds ratio; PPI, proton pump inhibitor; Prev, *Prevotella*.

^a^Defined as use of antibiotics for >48 hours within 12 weeks before surgery.

^b^ *P* value: Fisher exact test for categorical variables and Wilcoxon rank-sum test for continuous variables.*

^c^Corrected for type of surgery (included as random factor), *R*^2^ = 0.509. Numbers may not sum up to 100% due to excluded NAs.

### The ES-Firm-Prev Ratio is the Most Important Independent Risk Factor for SSIs Applying Both Machine Learning and Logistic Regression

**Figure 3.**
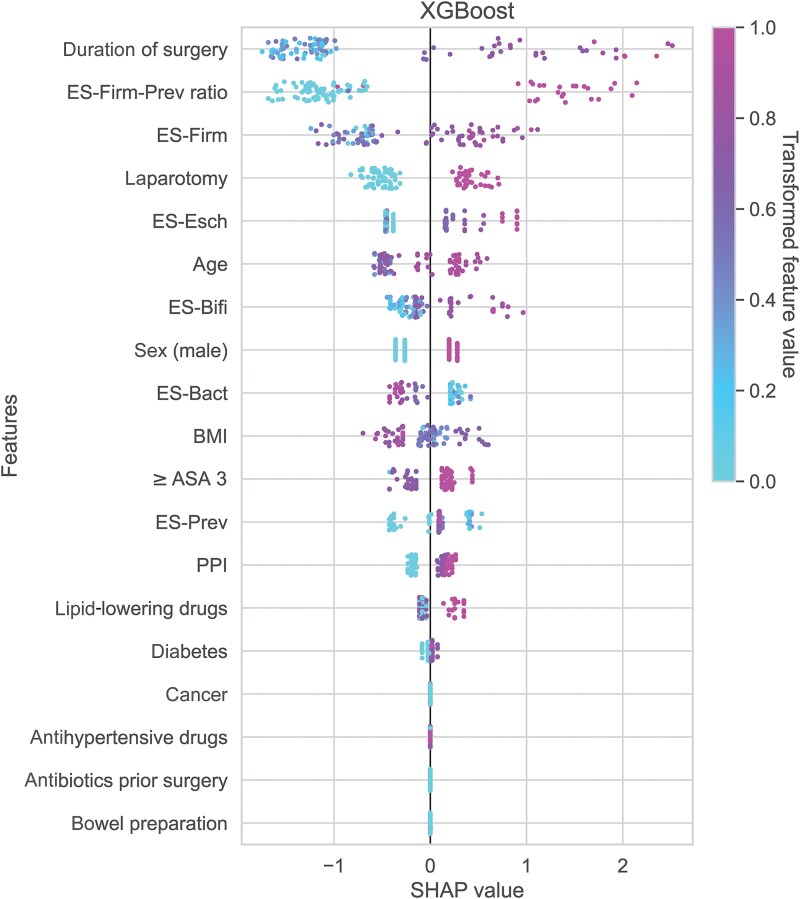
Feature importance analysis visualized by a SHAP density scatter plot. This visualization depicts the contribution of each feature to the XGBoost machine learning model's output. Abbreviations: ASA, American Society of Anesthesiologists score; Bact, *Bacteroides*; BMI, body mass index; Bifi, *Bifidobacterium*; ES, enterosignature; Esch, *Escherichia*; Firm, *Firmicutes*; PPI, proton pump inhibitor; Prev, *Prevotella*.

We trained 8 different machine learning classifiers on the ES-Firm-Prev ratio, the ESs, and clinical variables to predict SSIs (Supplementary Figure 4*A*). XGBoost had the highest overall predictive capacity (F-score) and was selected for further analysis. A feature importance analysis was performed to determine the factors mostly influencing the predictive performance of this model using a SHAP density scatter plot (Figure 3). The most important features to predict SSIs were surgery duration, ES-Firm-Prev ratio, ES-Firm, and whether open surgery (laparotomy) was performed. These positive correlations were also observed in the hierarchically clustered heatmap (Supplementary Figure 4*B*) and corresponded to the findings of the univariate analysis (Table 1). In a multivariable logistic regression including duration of surgery and ES-Firm-Prev ratio model-corrected for type of surgery, ES-Firm-Prev ratio was the most important independent risk factor associated with SSIs (odds ratio, 1.35; 95% CI, 1.09–1.66; *P* = .005) (Table 1, columns 5–6; Supplementary Figure 4C–F).

### ES-Firm-Prev Ratio, Which Increases With Age, Is Positively Associated With the Expression of Enzymes Metabolizing Simple Sugars and Starch

**Figure 4.**
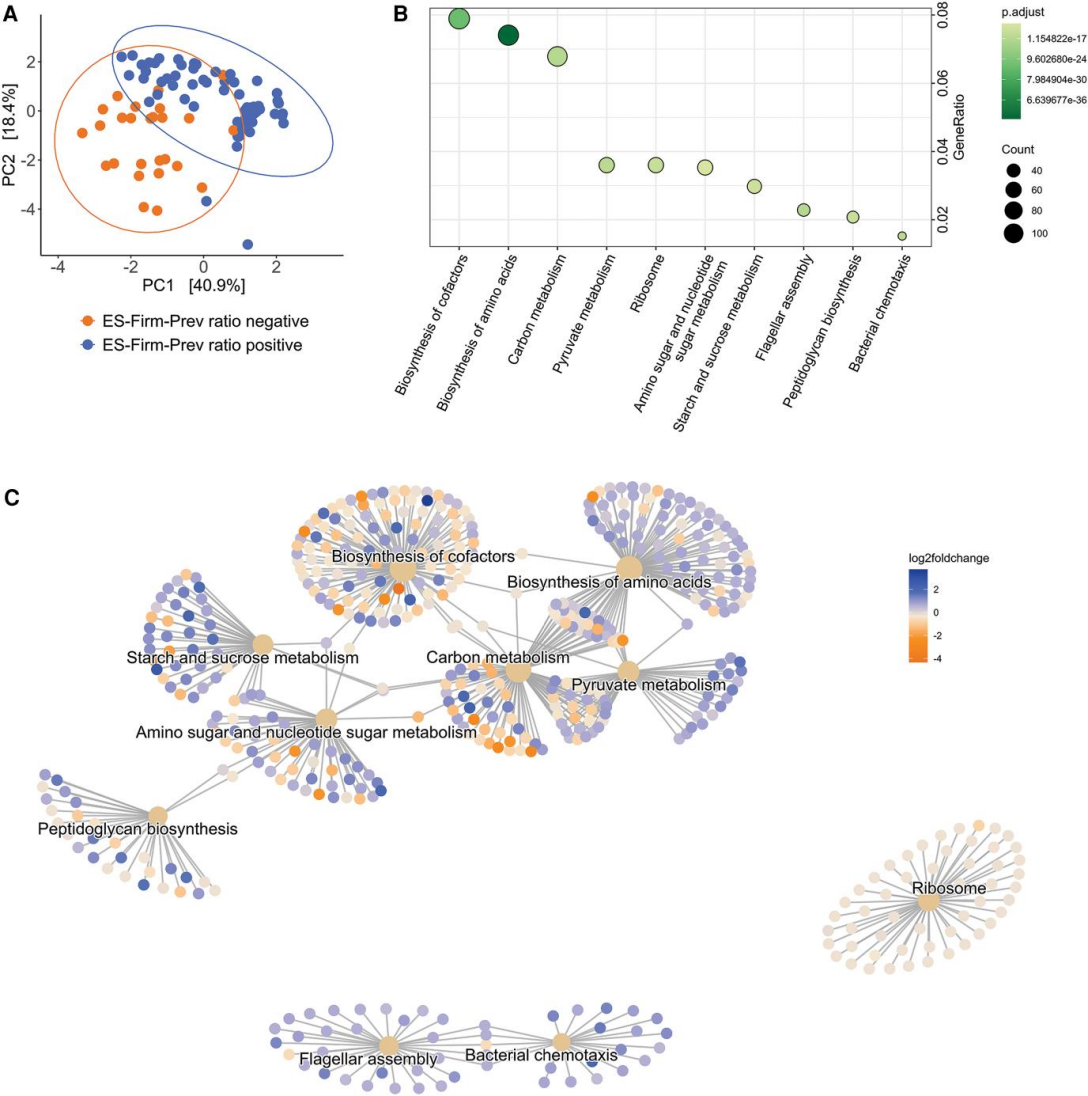
A, Principal component analysis of inferred KEGG orthologues using the PICRUSt2 pipeline, colored according to the ES-Firm-Prev ratio. B, Enrichment analysis of inferred KEGG orthologue abundances showing the most differentially expressed pathways between the 2 groups and (C) if the enzymes within the pathways are higher expressed in patients with a negative (orange) or a positive (blue) ES-Firm-Prev ratio. Abbreviations: ES, enterosignature; Firm, *Firmicutes*; PC, principal component; Prev, *Prevotella*.

The ES-Firm-Prev ratio and the age-related dynamics of ESs are shown in Supplementary Figure 5A. To understand why ES-Firm might be harmful and ES-Prev beneficial for patients, we compared the inferred metabolic profiles (PICRUSt2 [Phylogenetic Investigation of Communities by Reconstruction of Unobserved States]) [35] of positive and negative ES-Firm-Prev ratios. Clustering was significantly different between these 2 groups (Figure 4A). Enrichment analysis revealed key differences in the pathways shown in Figure 4B. Most enzymes belonging to the starch and sucrose or pyruvate metabolism pathways were higher expressed in samples with a positive ES-Firm-Prev ratio (Figure 4C;  Supplementary Figure 5B and C). In the biosynthesis of cofactors pathway, more enzymes, and especially those producing folate, were higher expressed in samples with a negative ES-Firm-Prev ratio (Figure 4C;  Supplementary Figure 5D).

## DISCUSSION

This is the first study applying the novel concept of ESs to uncover associations between preoperative intestinal microbiota composition and occurrence of SSIs. We used 50 high-quality genus-level bacterial taxa to calculate ESs and found a significant independent association between preoperative ES-Firm-Prev ratio and the occurrence of SSIs. Our combined analysis using machine learning and logistic regression underscores the potential of the ES-Firm-Prev ratio as a strong and valuable predictor of SSIs.

Studies assessing perioperative microbiota composition on the individual bacterial level and postoperative complications have shown inconsistent results [19–22, 36–38]. Some studies suggest a link between preoperative species including *Acinetobacter, Hafnia, Akkermansia muciniphila*, and *Fusobacterium* [19, 20, 22] and postoperative complications [19, 20] or SSIs [22]. Other studies have associated postoperative stool microbiota, including *Enterococci* [36, 37], *Enterobacteriales* [37], and multidrug-resistant organism (MDRO) colonization [38], with higher infection rates.

These studies often rely on stool samples [19–21, 36, 37], which vary significantly in timing and composition, or on ileal samples [22], which are only accessible in specific surgeries. In contrast, rectal mucosal swabs as applied in this study provide a standardized, site-specific snapshot of the preoperative rectal microbiota, minimizing variability and enhancing comparability.

While previous studies highlight the importance of perioperative intestinal microbiota in complication risk, their results are highly variable [19–22, 36–38]. Due to the vast individual variation in intestinal microbiota composition, previous results are likely to show study-specific differences in certain bacterial taxa without generalizable value, making it difficult to draw meaningful conclusions [19–22, 36, 37].

To overcome these limitations, we grouped individual bacterial taxa into 5 generalizable ESs dominated by *Bacteroides, Firmicutes, Prevotella, Bifidobacterium,* or *Escherichia*. We extended the original method by using 16S rRNA sequencing data from filter papers. This approach overcomes the limitations inherent to metagenomic sequencing, such as availability and cost. In accordance with Frioux et al., [24] who reported good generalizability of the ES model, even to non-Western cohorts, we had good model fit scores (median, 0.71) for our final cohort.

In our cohort, there was no association between sample or community diversity of individual bacterial taxa and SSIs. The community diversity of patients undergoing colorectal surgery was significantly different than that of individuals undergoing the other types of surgery, possibly due to preoperative bowel preparation [39]. However, only a very limited number of patients in our study received bowel preparation (Table 1). Building on the strengths of the ES model, however, we found a significant association between the ES-Firm-Prev ratio and occurrence of SSIs. We used a machine learning algorithm to assess the importance of ESs and other clinical factors in predicting SSIs. The 2 most important parameters were evaluated in a standard logistic regression model corrected for type of surgery. The significant association between the ES-Firm-Prev ratio and SSIs remained unaffected by clinical covariates. Unlike most of the well-established risk factors for SSIs such as duration of surgery, the ES-Firm-Prev ratio is a preoperatively assessable and potentially even adaptable risk factor for SSIs.

The ES-Firm-Prev ratio correlates with the patient's age, cancer diagnosis, laparotomy, duration of surgery, and preoperative antibiotic treatment. This indicates that older patients with cancer who have undergone preoperative treatments and require complex surgical interventions tend to have a shift toward increased ES-Firm-Prev ratio, which might explain why this patient group is especially at risk for SSIs. Our findings are in line with studies showing that loss of intestinal microbial stability in critically ill and intensively treated patients is associated with increased susceptibility to infections [14–16]. However, the ES-Firm-Prev ratio negatively correlated with increased American Society of Anesthesiologists score, diabetes, and body mass index (BMI). There were no significant differences in preoperative ES-Firm-Prev ratios between patients undergoing different types of abdominal surgery. So, the ES-Firm-Prev ratio seems not to be a direct indicator of overall health status. The positive correlation of the ES-Firm-Prev ratio with antibiotic use aligns with the findings of Frioux et al., who reported a negative correlation between antibiotic intake and ES-Prev [24]. Likewise the negative correlation between ES-Firm-Prev ratio and BMI and diabetes is consistent with results from Frioux et al., which reported a negative correlation of ES-Firm with patients’ BMI and HbA1c blood sugar values [24].

ES-Prev is predominantly composed of the *Prevotella* genus, and ES-Firm mainly of members of the *Firmicutes* phylum, as well as *Alistipes*. The role of *Prevotella* in human health is controversial. Some studies show a positive correlation between *Prevotella* and people with obesity [40, 41], while others report beneficial effects on metabolism such as improved glucose metabolism [42, 43]. The *Prevotella* genus is related to a plant-rich diet, which is high in fibers and complex polysaccharides, found in rural environments [44–46].

Most enzymes belonging to the pyruvate or starch and sucrose metabolism pathways are notably higher expressed in patients with a positive ES-Firm-Prev ratio. These readily available simple sugars might lead to a potentially harmful abundance of bacteria belonging to the ES-Firm. Conversely, enzymes belonging to the synthesis of cofactors pathway and especially the ones included in folate synthesis were notably higher expressed in patients with a negative ES-Firm-Prev ratio. Folate, which is important for various synthesis and repair mechanisms as well as for a strong immune system, might be absorbed and used by the host, potentially lowering risk for SSIs [47].

### Limitations

This study has several limitations. The single-center design with a relatively small sample size focusing on abdominal surgery restricts the generalizability of our findings. Although the ES model has high generalizability, 24 samples had a low model fit and could not be assigned to ESs. In the logistic regression analysis, we included the 2 most important parameters identified by the machine learning model and adjusted for the type of surgery. While this reduces the risk of overfitting, we may have missed other important confounders. Despite being trained on different data sets, the ESs may not fully represent the diversity of the human intestine. To our knowledge, this is the largest cohort of standardized preoperative intestinal samples assessing SSI risk. However, the findings should be confirmed in larger multicenter cohorts. Additionally, while the correlation matrix and functional analysis may offer insights into the mechanisms linking the ES-Firm-Prev ratio and SSIs, further mechanistic exploration is needed in future studies.

## CONCLUSIONS

Our results imply that the ES-Firm-Prev ratio may serve as a preoperative biomarker to identify patients at risk for SSIs. This finding could allow clinicians to implement personalized preventive measures, potentially reducing postoperative SSIs.

## Supplementary Material

ofaf549_Supplementary_Data

## Supplementary Data

Supplementary materials are available at *Open Forum Infectious Diseases* online. Consisting of data provided by the authors to benefit the reader, the posted materials are not copyedited and are the sole responsibility of the authors, so questions or comments should be addressed to the corresponding author.
