# Supplementary material for: Preoperative Enterosignatures Predict Surgical Site Infections After Abdominal Surgery
Source: Open Forum Infect Dis. 2025 Sep 3;12(9):ofaf549. doi: 10.1093/ofid/ofaf549 (PMC12456171; doi:10.1093/ofid/ofaf549)
Supplement: ofaf549_Supplementary_Data [file ofaf549_supplementary_data.docx]

**Preoperative Enterosignatures Predict Surgical Site Infections After Abdominal Surgery**

Simone N. Zwicky, MD*^1^, Daniel Spari, MD,PhD*^1^, Daniel Rodjakovic, MD^1^, Hugo Guillen-Ramirez, PhD^1^, Bahtiyar Yilmaz, PhD^1^, Guido Beldi, MD^2^

- ^1^Department of Visceral Surgery and Medicine, Inselspital, Bern University Hospital, University of Bern, Freiburgstrasse 18, 3010, Bern, Switzerland.
- ^2^**Corresponding author:** Department of Visceral Surgery and Medicine, Inselspital, Bern University Hospital, University of Bern, Freiburgstrasse 18, 3010, Bern, Switzerland. Phone +41 31 632 82 75, guido.beldi@insel.ch, ORCID ID: 0000-0002-9914-3807.

**Supplementary Materials - Index**

| **Supplementary Figures** |  |
| --- | --- |
| SFigure 1. Assessment of the generalizability of the sampling method | *pag. 2* |
| SFigure 2. Legend of genus-level taxonomy of all patients from Figure 1D | *pag. 3* |
| SFigure3. ES-Firm-Prev ratio with type of surgery & relative abundances of ESs of noSSI vs SSI patients | *pag. 4* |
| SFigure4. Model performances, hierarchically clustered heatmap using the final model and important features projected on community diversity | *pag. 5* |
| SFigure5. Dynamics of ESs over age and in-depth analysis of starch and sucrose, pyruvate and biosynthesis of cofactors pathways | *pag. 6* |

**Supplementary Figures**

**SFigure 1. Assessment of the generalizability of the sampling method**
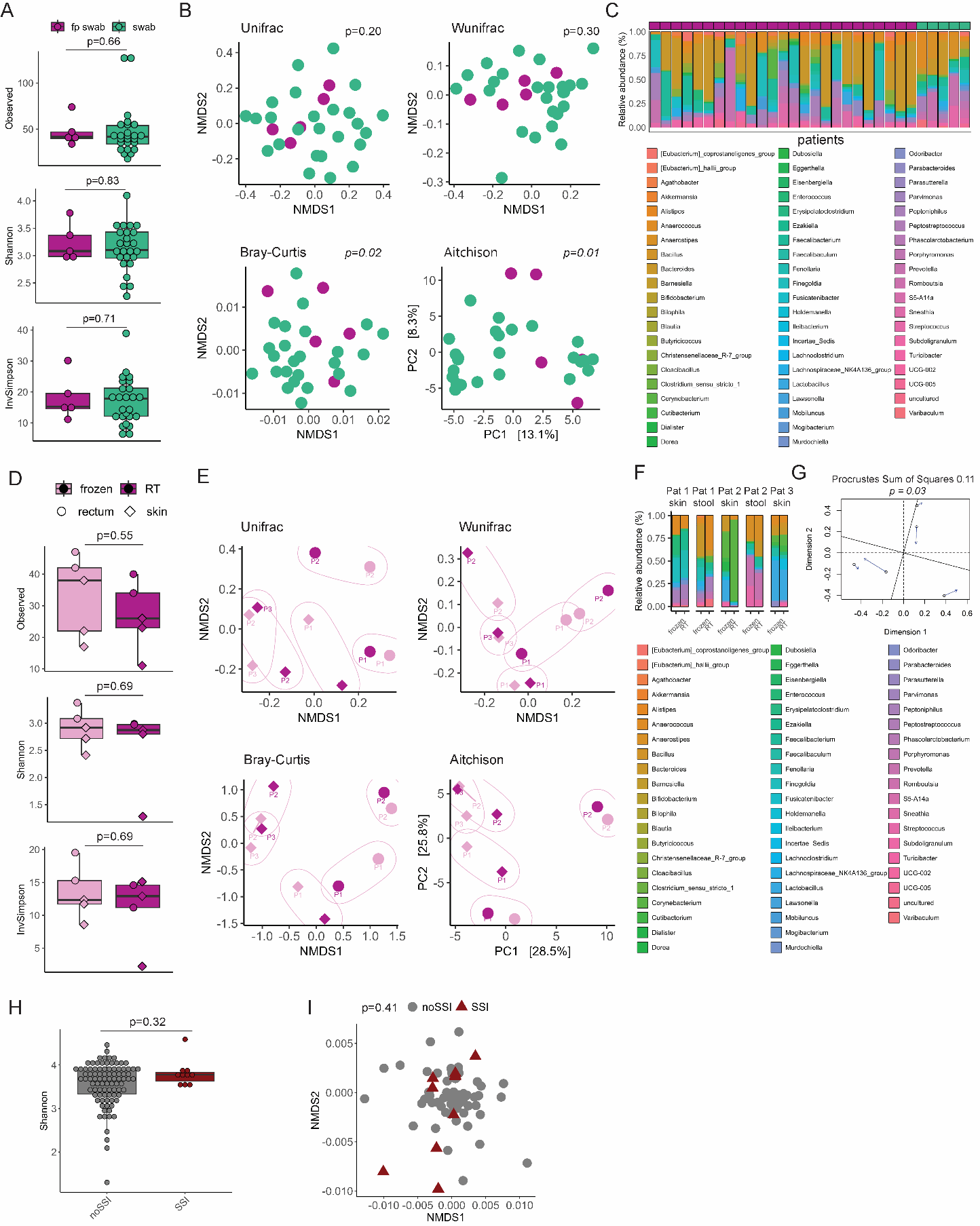


A) Sample diversity (alpha diversity, Observed; Shannon; InvSimpson), B) community diversity (beta diversity, Unifrac; Wunifrac; Bray-Curtis; Aitchison) and C) genus level taxonomy of swabs streaked on filter paper compared to directly frozen swabs. D) Sample diversity (alpha diversity, Observed; Shannon; InvSimpson), E) community diversity (beta diversity, Unifrac; Wunifrac; Bray-Curtis; Aitchison), F) genus level taxonomy and G) Procrustes analysis of swabs streaked on filter paper and stored at RT compared to storage at -80°C. H) Sample diversity (alpha diversity, Shannon) and I) community diversity (beta diversity, Bray-Curtis) from rectal swabs of patients without SSI compared to patients with SSI.

Abbreviations: fp, filter paper; RT, room temperature; SSI, surgical site infection; NMDS, non-metric multidimensional scaling; PC, principal component.

**SFigure 2. Legend of genus-level taxonomy of all patients from Figure 1D**


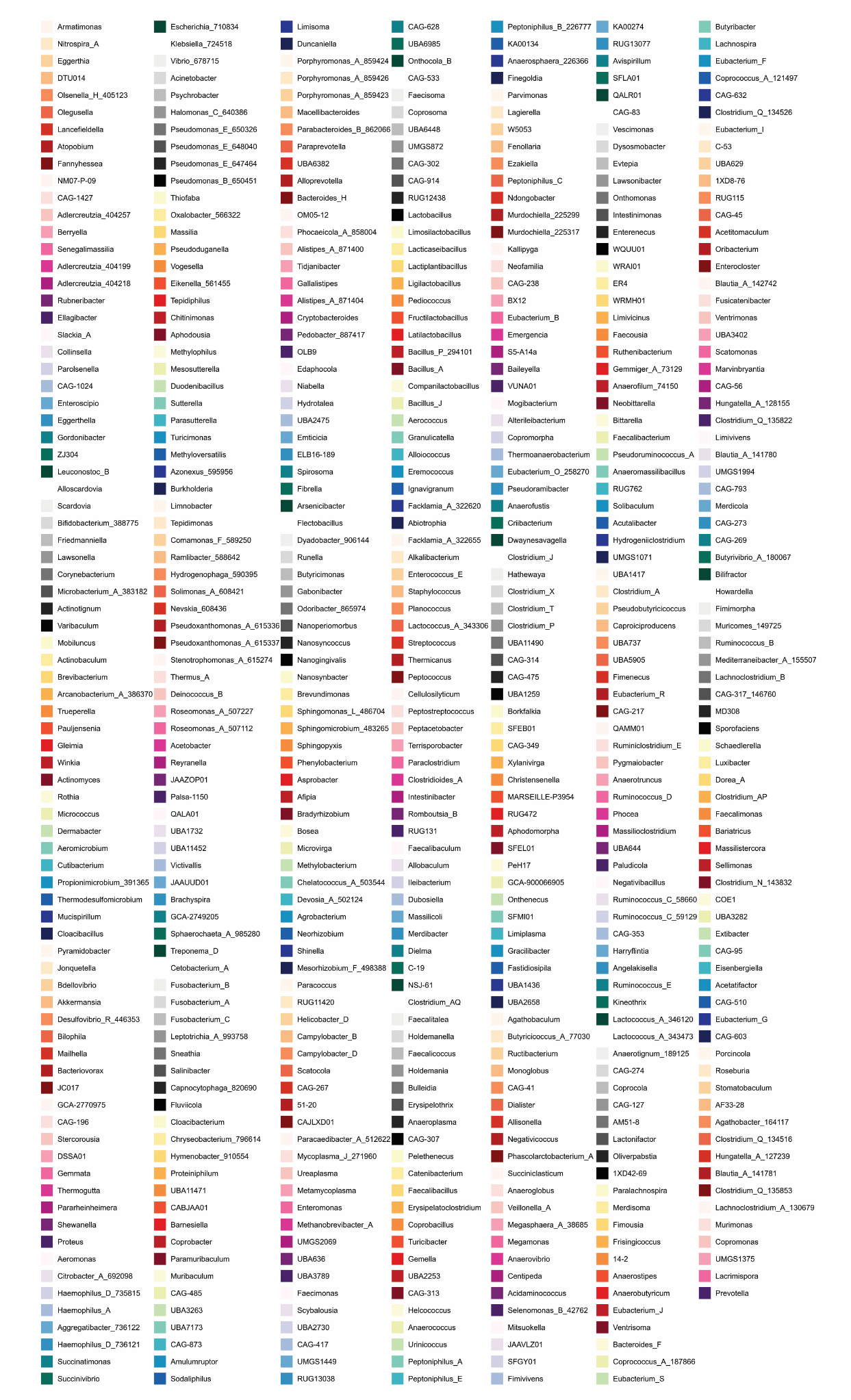


**SFigure3. ES-Firm-Prev ratio with type of surgery & relative abundances of ESs of noSSI vs SSI patients**


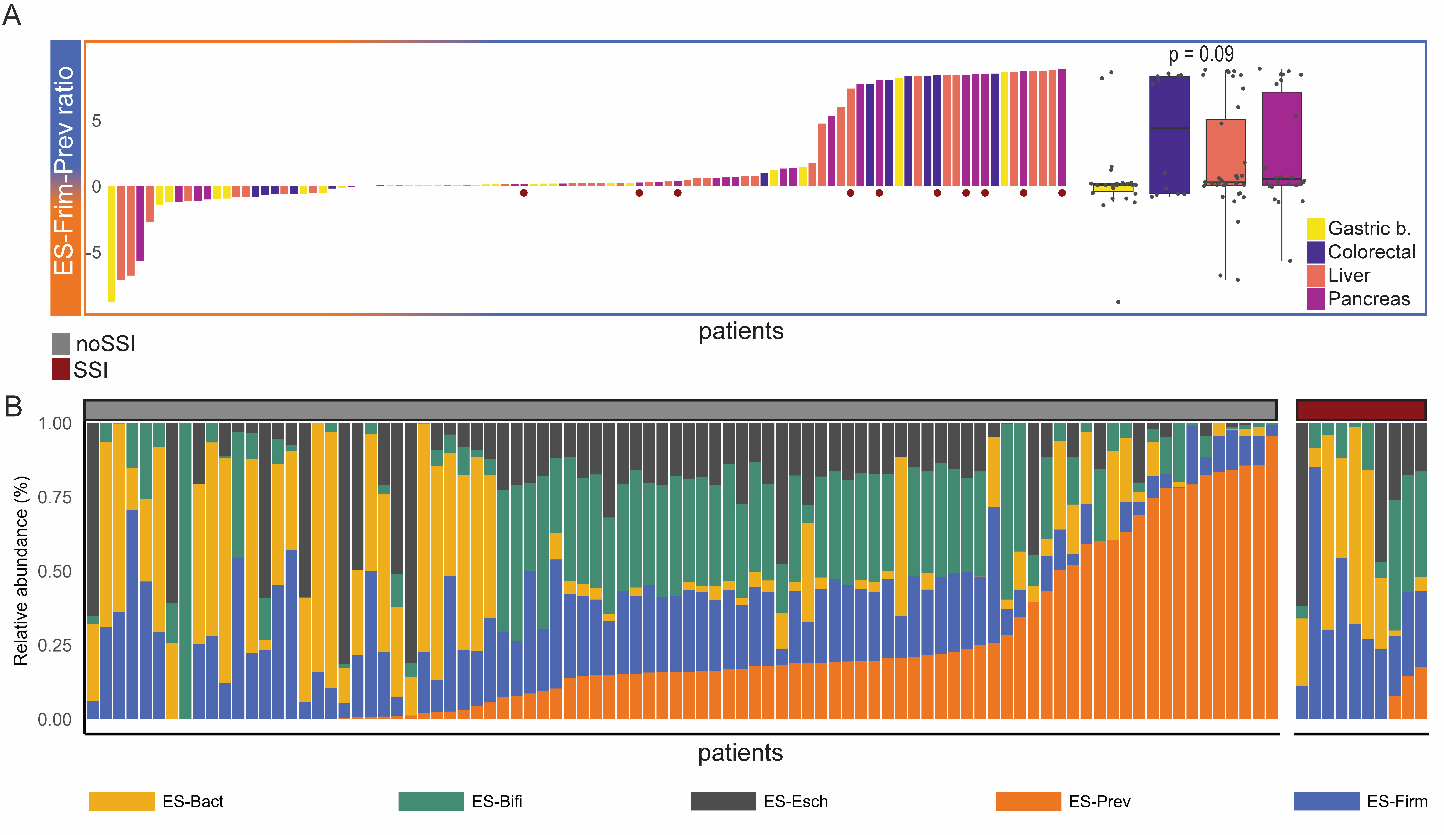


A) Comparison of ES-Firm-Prev ratio among the different types of surgery using a Kruskal–Wallis one-way ANOVA. B) Relative abundance of ESs in the noSSI versus SSI patients.

Abbreviations: Gastric b., Gastric bypass; ES, Enterosignature; Bact, Bacteroides; Bifi, Bifidobacterium; Esch, Escherichia; Prev, Prevotella; Firm, Firmicutes; SSI, surgical site infection

**SFigure4. Model performances, hierarchically clustered heatmap using the final model and important features projected on community diversity**

**
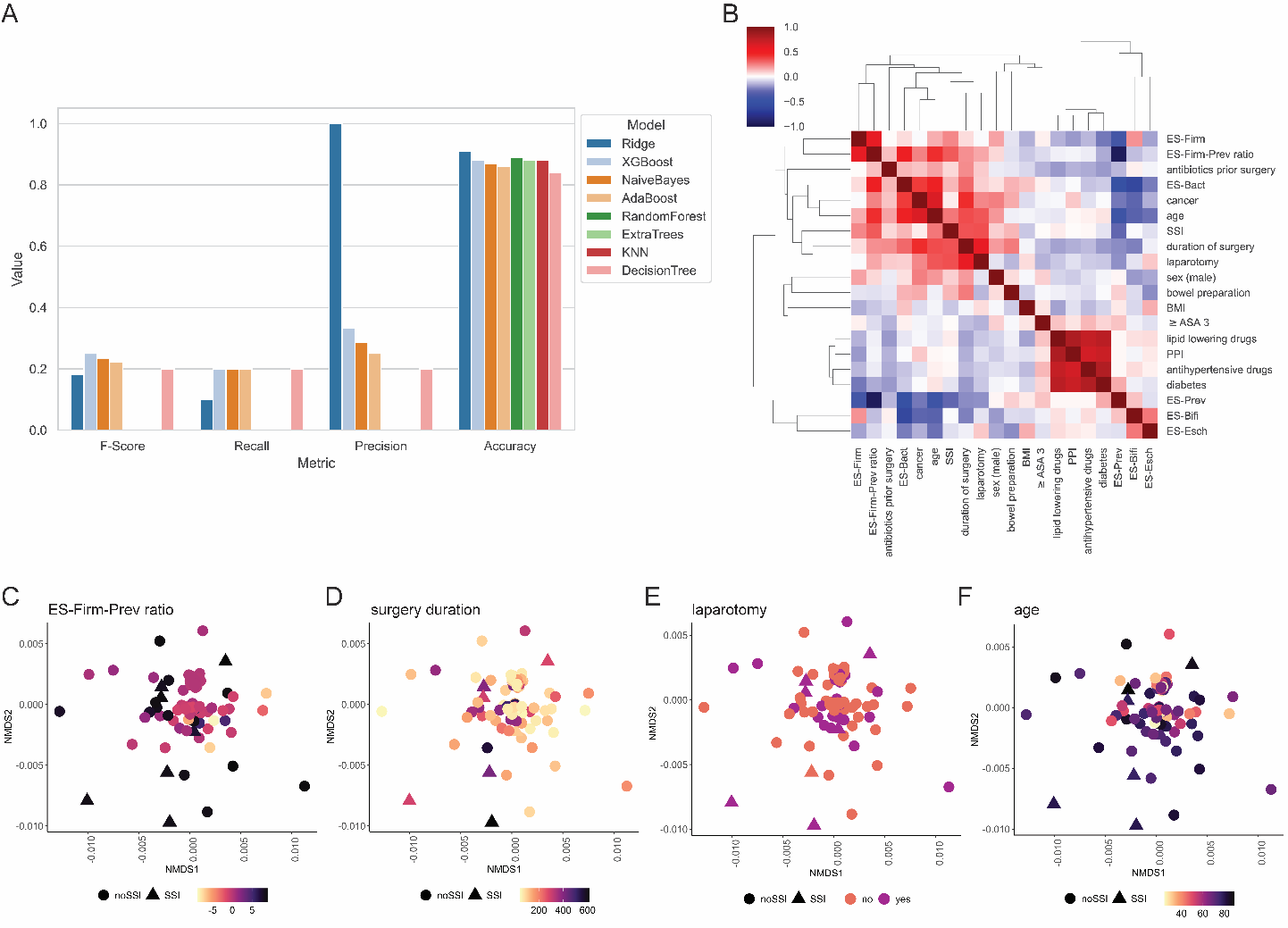
**

A) Barplot of the performances of the machine learning classifiers (F-score, Recall, Precision, Accuracy). B) Hierarchically clustered heatmap of correlations between patient characteristics, ES-Firm-Prev ratio and ESs. C-F) Projection of most important features on community diversity (beta diversity, Bray-Curtis).

Abbreviations: KNN, K-nearest neighbors; BMI, body mass index; PPI, proton pump inhibitor; ASA, American Society of Anesthesiologists score; ES, Enterosignature; Bact, Bacteroides; Bifi, Bifidobacterium; Esch, Escherichia; Prev, Prevotella; Firm, Firmicutes; SSI, surgical site infection.

**SFigure5. Dynamics of ESs over age and in-depth analysis of starch and sucrose, pyruvate and biosynthesis of cofactors pathways**

**
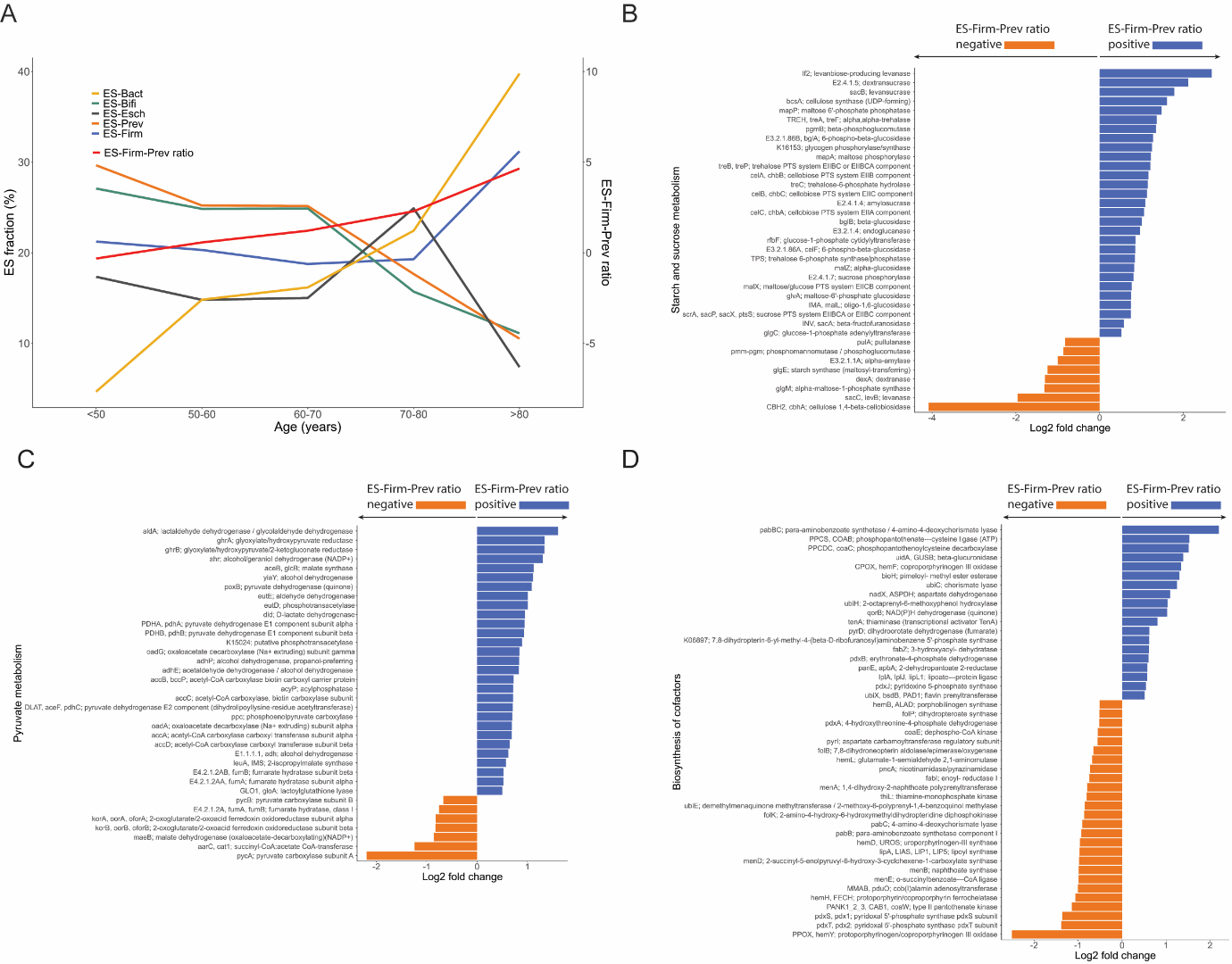
**

A) ES-Firm-Prev ratio and fractions of ESs over age. B-D) In-dept analysis of differences (log2 fold changes (>0.5 | < -0.5)) between patients with a negative and a positive ES-Firm-Prev ratio in the starch and sucrose, the pyruvate and the biosynthesis of cofactors pathways.

Abbreviations: ES, Enterosignature; Bact, Bacteroides; Bifi, Bifidobacterium; Esch, Escherichia; Prev, Prevotella; Firm, Firmicutes.
